# Diversity of bacteria and archaea in the rhizosphere of bioenergy crop *Jatropha curcas*

**DOI:** 10.1007/s13205-016-0546-z

**Published:** 2016-12-02

**Authors:** Garima Dubey, Bharati Kollah, Vijay Kumar Gour, Arvind Kumar Shukla, Santosh Ranjan Mohanty

**Affiliations:** 1 Indian Institute of Soil Science, Nabibagh, Bhopal, 462038 India; 2 Department of Plant Breeding and Genetics, J.N. Agricultural University, Krishinagar, Jabalpur, 482004 India

**Keywords:** Diversity, Bacteria, Archaea, Rhizosphere, Bioenergy crop, *Jatropha curcas*

## Abstract

Plant-microbial interaction in rhizosphere plays vital role in shaping plant’s growth and ecosystem function. Most of the rhizospheric microbial diversity studies are restricted to bacteria. In natural ecosystem, archaea also constitutes a major component of the microbial population. However, their diversity is less known compared to bacteria. Experiments were carried out to examine diversity of bacteria and archaea in the rhizosphere of bioenergy crop *Jatropha curcas* (*J. curcas*). Samples were collected from three locations varying widely in the soil physico-chemical properties. Diversity was estimated by terminal restriction fragment length polymorphism (TRFLP) targeting 16S rRNA gene of bacteria and archaea. Fifteen bacterial and 17 archaeal terminal restriction fragments (TRFs) were retrieved from *J. curcas* rhizosphere. Bacterial indicative TRFs were *Actinobacteria, Firmicutes, Acidobacteria, Verrumicrobiaceae,* and *Chlroflexi*. Major archaeal TRFs were crenarchaeota, and euryarchaeota. In case of bacteria, relative fluorescence was low for TRF160 and high for TRF51, TRF 420. Similarly, for archaea relative fluorescence of TRF 218, and TRF 282 was low and high for TRF 278, TRF468 and TRF93. Principal component analysis (PCA) of bacterial TRFs designated PC 1 with 46.83% of variation and PC2 with 31.07% variation. Archaeal TRFs designated 90.94% of variation by PC1 and 9.05% by PC2. Simpson index varied from 0.530 to 0.880 and Shannon index from 1.462 to 3.139 for bacteria. For archaea, Simpson index varied from 0.855 to 0.897 and Shannon index varied from 3.027 to 3.155. Study concluded that rhizosphere of *J. curcas* constituted of diverse set of both bacteria and archaea, which might have promising plant growth promoting activities.

## Introduction

Bioenergy crop *Jatropha curcas* (*J. curcas*) is a member of Euphorbeaceae family. Recently, it is promoted worldwide for bioenergy production, soil health improvement, climate change mitigation, carbon sequestration and socio economic improvement (Islam et al. 2014). *J. curcas* survives under limited nutrient and harsh environmental condition. *J. curcas* adapt to grow under salt stress, high temperature. Because of these growth abilities, *J. curcas* has been used for soil reclamation. Its rhizosphere harbors myriad of microbial community those regulate nutrient uptake and adaptation to extreme environment. Many heterotrophs, N_2_ fixers, and P solubilizers inhabit in its rhizosphere (Mohanty et al. 2012). Rhizosphere of Jatropha possesses diverse group of arbuscular mycorrhizal fungi (AMF), actinomycetes, and many plant growth-promoting rhizobacteria (PGPR) (Pérez and Schenck 1990). Plant growth-promoting gamma-proteobacterium, *Enterobacter cancerogenus* has also been isolated from the rhizosphere of *J. curcas.* These microbial species produce growth hormone ACC (1-amino-cyclopropane-1-carboxylate) deaminase, phytases, auxin, IAA (indole acetic acid), siderophores, ammonia and solubilize phosphate. Like *J. curcas*, most of the plants have numerous PGPRs in their rhizosphere, but they often fail to grow under challenging environment. However, it is unclear how *J. curcas* adapts to extreme (water and nutrient limitation) conditions. We hypothesize that *J. curcas* harbors certain microbial groups in its rhizosphere which might have different plant-microbial interaction than commonly known. Possibly archaea predominate in *J. curcas* rhizosphere, because archaea are known to occur in challenging environments. The present study was undertaken to reveal the diversity of bacteria and archaea in the rhizosphere of *J. curcas*.

## Materials and methods

### Site characterization

Sampling was carried out during July 2014 from three sites located at the Jabalpur, Noida, and Bhopal (Table 1). *Jatropha curcas* is being grown in these locations since 2006. Samples from Bhopal was located near the village Gunga, Bhopal, Madhya Pradesh, India (23°45′N latitude, 77°36′E longitude and 485 m above mean sea level). Geographically, this location has semi-arid and sub-tropical climate. It has dry summer and cold winter with a mean annual air temperature of 25 °C, rainfall 1208 mm and humidity 56%. It experiences southwestern monsoon rains in July–September. The experimental soil belongs to vertisols that is, Hypothermic family of Typic Haplusterts popularly known as “black cotton soil”. Samples from Jabalpur were collected from experimental farm of Jawaharlal Nehru Krishi Vishwya Vidyalaya, Jabalpur (24°30′N latitude, 80°15′E longitude and 306.06 m above from sea level). The soil of experimental field was sandy clay loam. Soil samples were also collected from Noida, Uttar Pradesh (25°53′N latitude, 77°39′E longitude and about 656 m above from sea level). The soil characteristics of the experimental site before start of the study were analyzed and presented in Table 1. The soils were obtained by removing the top soil (~1–2 cm) and collecting the next 10 cm with a shovel near plant’s root (Mohanty et al. 2015). Three randomly collected individual samples were mixed to make a composite sample. Soils were stored in plastic bags at 4 °C to prevent moisture loss and were used within 48 h of collection.

**Table 1:** Physico chemical properties of soils collected from different sampling sites

Location	Textural class	pH (1:2.5 soil to water)	EC (1:2.5 soil to water, dSm^−1^)	CEC (cmol (p+) kg^−1^)	Organic C (g kg^−1^)	Total *N* (g kg^−1^)	Sand (%)	Silt (%)	Clay (%)
Bhopal	Heavy Clayey	7.5	0.43	44.5	5.7	0.61	15.2	30.3	54.5
Jabalpur	Clayey Typic Haplustert	7.2	0.22	9.8	4.5	0.22	25.3	17.9	56.8
Noida	Sandy clay loam	8.1	0.24	10.6	6.8	0.57	47.4	27.2	24.8

### DNA extraction from soil and PCR amplification

Freshly collected rhizospheric soils of 0.5 g were used to extract DNA using ultraclean DNA extraction kit (MoBio, USA) as described by manufacturer. The extracted DNA was dissolved in 50 μl TE buffer (10 mM, pH 8.0) and stored at −20 °C until further analysis. The PCR was performed in a total volume of 50 μl containing 20 ng of DNA template, 1U of Taq DNA polymerase (NEB, USA), 0.4 μM of each primer, and 1× PCR buffer (PCR Buffer II, Applied biosystems, CA, USA). The bacterial primers were 8f (5′-AGAG TTT GAT CCT GGC TCA G-3′) and 518r (5′-CTC CTA CGG GAG GCA GCA G-3′). The Achaeal primers were 109F (5′-ACK GCT CAG TAA CAC GT-3′) and 915R (5′-GTG CTC CCC CGC CAA TTC CT-3′). Forward primers were labeled with 6-Carboxyfluorescein (FAM) at the 5′ end. Thermal cycling for bacterial 16 s rRNA was carried out in a PCR instrument (Step one plus, ABI) by an initial denaturizing step at 94 °C for 4 m, 35 cycles of 94 °C for 1 m, 50 °C (bacteria) or 56 °C (archaea) for 30 s, 72 °C for 45 s; final extension carried out at 72 °C for 5 m. The presence and sizes of the PCR amplification products were determined by agarose (1%) gel electrophoresis. PCR products were purified from the reaction mix using the PCR purification kit (Axygen, USA).

### Terminal restriction fragment length polymorphism (T-RFLP) analysis and phylogenetic affiliation

Efficiency of the T-RFLP depends on the coverage of the restriction enzymes. Efficiency of three restriction enzymes (Msp, Rsa and Alu) evaluated to identify best restriction enzyme for diversity analysis. Bacterial and archaeal 16SrRNA gene sequences were downloaded from GenBank. Web based tools (MiCA: T-RFLP Analysis APLAUS+) were used for in silico analysis (Shyu et al. 2007). Virtual amplification and digestion was carried out with respective primers and different restriction enzymes as per instructions. Based on the number of terminal restriction fragments (TRFs) and coverage restriction enzymes were finalized. In silico analysis identified RsaI (GT^AC) as best restriction enzyme for bacteria and Alu (AG^CT) for archaea. Amplicons were digested by respective restriction enzymes at 37 °C for 3 h and inactivated at 65 °C for 20 min as per manufacturer’s instruction (New England Biolabs, USA). The digested products were sent for fragment analysis to Macrogen Inc, Korea (www.macrogen.com). Briefly, aliquots of 10 μl were mixed with 15 μl HiDi formamide (Applied Biosystems) and 0.3 μl of the internal DNA fragment length standard (500 LIZ, Applied Biosystems). Fragment analysis was conducted on a 3730xl DNA analyzer (Applied Biosystems). T-RFs were determined using the GeneScan software package (version 1.0, Applied Biosystems), Fragments between 50 and 500 bp were included in the analysis. Peak area data were normalized for uniform distribution. Each peak expressed as a percentage of the total peak area in the profile. Peaks comprising <1% of the total area were removed from the analysis (Mohanty et al. 2007). Phylogenetic assignments were attempted from T-RFLP patterns using the web-based program PAT (MiCA) (Shyu et al. 2007). Although identifications are tentative, however, this in silico approach has been successfully validated with clone libraries in other studies (Mohanty et al. 2007; Yi et al. 2009; Tipayno et al. 2012). It allowed retrieval not only of the composition of bacteria down to the genus level, but also of many ribotypes related to a specific organism from environmental samples (Rösch and Bothe 2005).

### AMMI model, PCA, diversity and cluster analysis

Raw T-RFLP profiles data from Peak Scanner were pre-treated using the online software T-REX to find out the most prominent TRFs (Culman et al. 2008). Microbial diversity was calculated from the relative fluorescence intensity of TRFs. The environmental variable was sampling location. Three replicate samples were computed with threshold limit 3 bp (fragments of 3 bp difference will be grouped as one representative) using the web-based tool (T-Rex). Output files were two way analysis of variance (ANOVA), additive main effects and multiplicative interaction (AMMI) model, and the abundance graph. The AMMI model is used to differentiate the effect of environmental factors on the TRFLP pattern (Culman et al. 2008). Multivariate T-RFLP data analysis carried out after organizing the data into a species (T-RFs)× samples matrix. This matrix contain three distinct sources of variation: (1) main effects for T-RFs, also called operational taxonomic units (OTUs); (2) main effects for Environments (E), also called treatments (source of sampling) and (3) interaction effects for T-RF × Environment (T × E). The method generally analyzed all three sources of variation simultaneously. To identify the major variables (TRFs) and the complexity of interaction with the environments, Principal Component Analysis (PCA) was carried out after ln transformation and normalization of data. PCA often reveals previously unsuspected correlations among the variables, thereby allowing interpretations that would not otherwise be apparent (Johnson and Wichern 2002). PCA analysis was interpreted graphically by constructing biplots, with the original variables drawn as vectors that summarize the correlation among the variables. PCA biplots are a convenient way of mapping the original variables, because the angles between the variables express their level of the correlation. Biplot was made using the values of three replicated observations. Average TRF data were subjected to cluster analysis using the Euclidean distance measure. Dendrogram was constructed using an un-weighted pair-group method with arithmetic mean. Diversity statistics were calculated from normalized profiles of individual samples using the number and area of peaks in each profile as representations of the number and relative abundance of phylotypes (Dunbar et al. 2001). Phylotype richness (S) was calculated as the total number of distinct TRF sizes in a profile. The Shannon diversity index, Simpson index and Evenness estimated using statistical package PAST (ver 2.12). All other analysis were carried out using agricolae and vegan package of the statistical software R ver 2.15.1 (Oksanen et al. 2007; De Mendiburu 2014).

## Results

### TRFLP analysis of bacteria and archaea

**Fig. 1:**
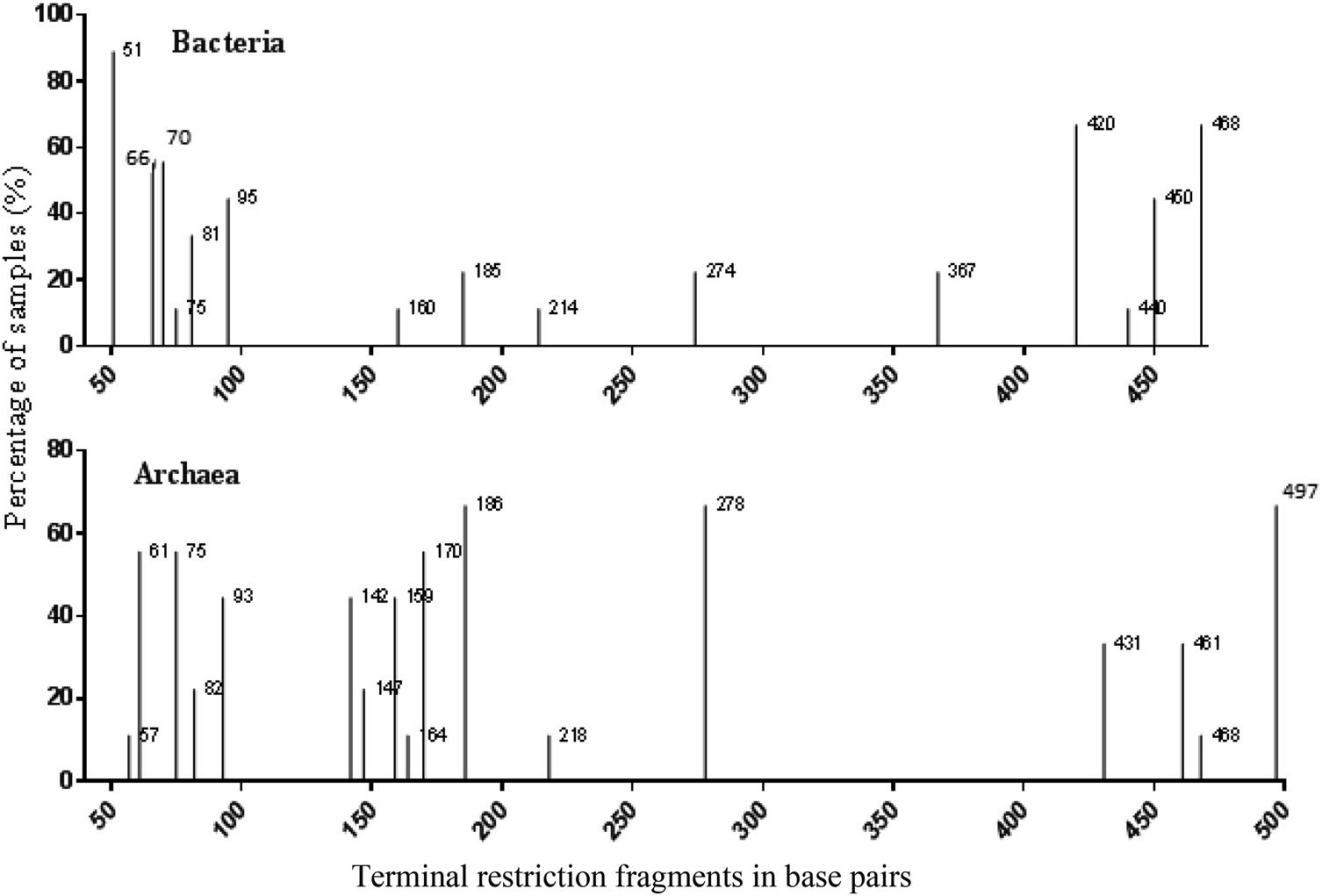
Terminal restriction fragment of bacteria and archaea prevalence in the rhizosphere of the Jatropha curcas. *X axis* represents base pairs, *Y axis* represents TRFs abundance in the rhizospheric samples

There were altogether 15 bacterial TRFs retrieved from the rhizospheric samples of *J. curcas* (Fig. 1). Fluorescence intensity of bacteria varied from 11 to 89%. Among those TRF75, TRF160, TRF214, and TRF499 had lowest intensity, while, TRF51 had highest intensity. Similarly, 17 archaeal TRFs were obtained from *J. curcas* rhizosphere. Fluorescence intensity of these TRFs varied from 11.11 to 67%. TRF57, TRF164 and TRF468 had lowest and TRF186, TRF278, and TRF 497 exhibited highest fluorescence intensity.

### Relative abundance of bacteria and archaeal TRFs

**Fig. 2:**
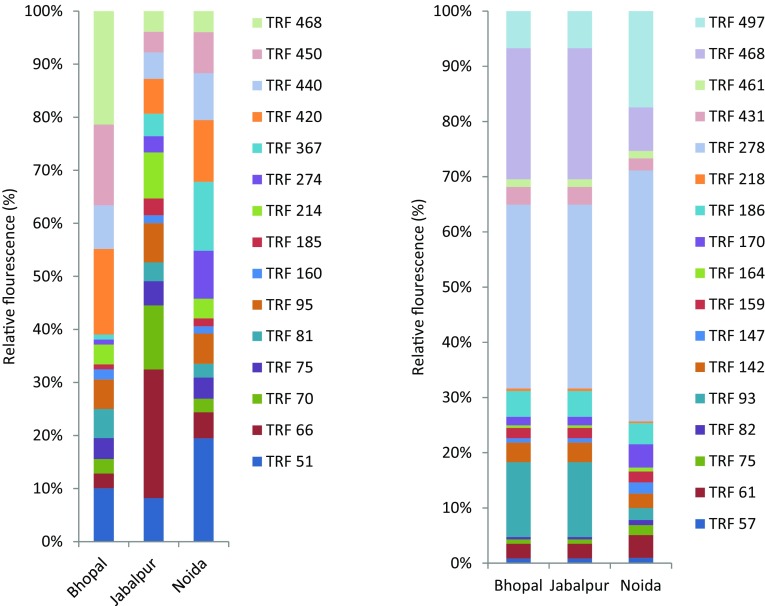
Relative fluorescence of bacterial (*left*) and archaeal (*right*) ribotypes (TRFs) in rhizosphere of *Jatropha curcas* collected from different locations. *X axis* represents sampling locations (Bhopal, Jabalpur and Noida). *Y axis* represent relative fluorescence of the TRFs. Each *data points* are average of three replicated observations

The relative abundance of the bacteria and archaea was evaluated as fluorescence of individual peaks in relation to the total fluorescence (Fig. 2). Relative fluorescence of the bacterial TRFs varied from 0.94% (TRF274, TRF367) to 21.38% (TRF468) in samples of Bhopal. In case of Jabalpur, relative fluorescence intensity ranged from 1.46% (TRF160) to 24.24% (TRF66). Samples from Noida exhibited relative fluorescence from 1.36% (TRF160) to 19.47% (TRF51). Like bacteria, relative fluorescence of archaea also followed similar pattern in the samples. Relative fluorescence of archaeal TRFs ranged from 0.44% (TRF82) to 23.75% (TRF468) in Bhopal. Jabalpur samples exhibited relative fluorescence from 0.43% (TRF218) to 33.32% (TRF278). In case of Noida, the relative fluorescence varied from 0.30% (TRF 218) to 17.42% (TRF497).

### Taxonomic assignment

The affiliation of TRFs representing different taxonomic units of bacteria and archaea is presented in Table 2. Major bacterial species were belonged to *Actinobacteria, Firmicutes, Proteobacteria, Bacteroidetes, Chloroflexi, Verrumicrobia,* and *Acidobacteria*. Three 3 TRFs (TRF51, TRF 75, TRF214) represented actinobacteria. Five TRFs (TRF66, TRF95, TRF440, TRF450, and TRF468) represented Firmicutes. Two TRFs (TRF 160, TRF 420) represented phyla Proteobacteria. The TRF367 represented *Acidobacteria* while TRF185 represented *Chloroflexi*. Archaeal TRFs mostly represented the uncultured species. Out of the 17 TRFs, 11 were uncultured archaea. Those were TRF57, TRF61, TRF82, TRF147, TRF159, TRF164, TRF 218, TRF 278, TRF 431, TRF 461, and TRF497. The euryarchaeal species were represented by TRF 75, TRF170, and TRF468. The crenarchaeota were represented by TRF93, TRF142, and TRF186.

**Table 2:** Tentative major bacterial and archaeal TRFs and their taxonomic affiliation

Bacteria		Archaea	
TRFs	Affiliation	TRFs	Affiliation
51	Bifidobacteriaceae	57	Uncultured archaea
66	Lactobacillaceae	61	Uncultured archaea
70	Moraxellaceae	75	Methanomicrobiaceae
75	Nocardiaceae	82	Uncultured archaea
81	Cytophagaceae	82	Uncultured archaea
95	Clostridiaceae	93	Crenarchaeota
160	Acetobacteriaceae	142	Crenarchaeota
185	*Chloroflexi*	147	Uncultured archaea
214	Micrococcaceae	159	Uncultured archaea
274	*Verumicrobia*	164	Uncultured archaea
367	*Acidobacteria*	170	Euryarchaeota
420	Sphingomonaceae	186	Crenarchaeota
440	Clostridiaceae	218	Uncultured archaea
450	*Bacillus*	278	Uncultured archaea
468	*Bacillus*	431	Uncultured archaea
		461	Uncultured archaea

TRFs are presented as base pairs

### Diversity analysis

Irrespective of sampling sites, the diversity of archaea was higher than the bacteria (Table 3). The Simpson (D) index of bacteria varied from 0.87 to 0.53. Archaeal simpson index varied in the range of 0.855–0.897 with lowest in Jabalpur and highest in Bhopal. Shannon index (H) for bacteria was high in Bhopal and low in Jabalpur. Similarly, Shannon index for Archaea was in the range of 3.027–3.155.

**Table 3:** Diversity indices of bacteria and archaea in rhizosphere of Jatropha curcas sampled from different locations

Location	Simpson (D)		Shannon (H)		Evennesss (H/S)	
	Bacteria	Archaea	Bacteria	Archaea	Bacteria	Archaea
Bhopal	0.880 ± 0.04	0.897 ± 0.025	3.139 ± 0.68	3.111 ± 0.23	0.360 ± 0.61	0.311 ± 0.024
Jabalpur	0.530 ± 0.05	0.855 ± 0.017	1.462 ± 0.29	3.027 ± 0.25	0.139 ± 0.25	0.226 ± 0.033
Noida	0.875 ± 0.08	0.873 ± 0.021	3.071 ± 0.074	3.155 ± 0.35	0.326 ± 0.47	0.286 ± 0.057

### PCA and cluster analysis

**Fig. 3:**
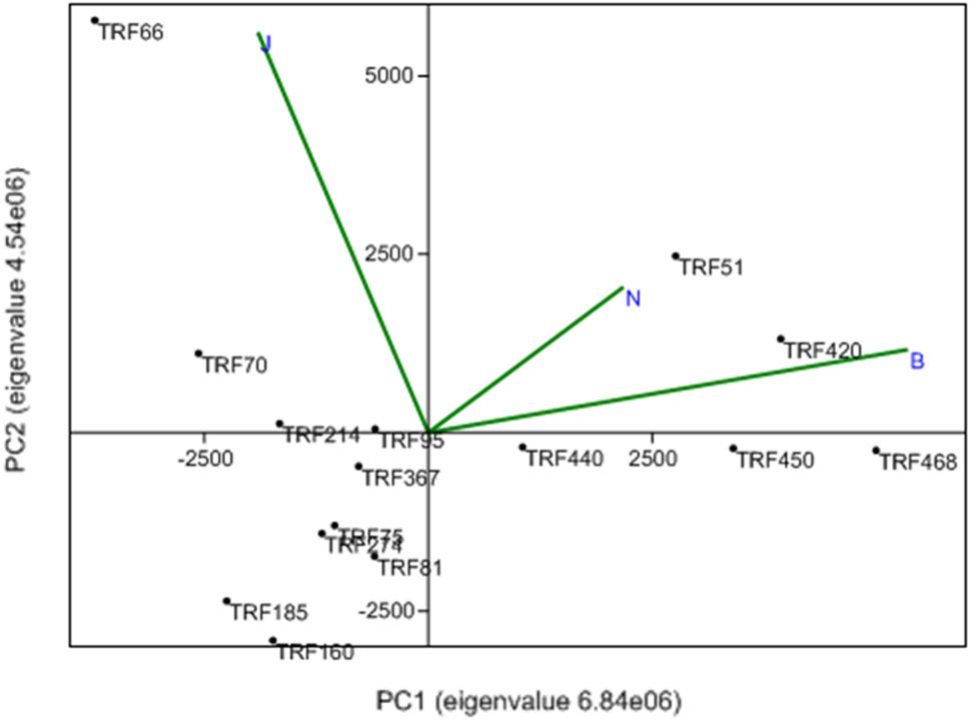
Principal component analysis (PCA) of bacterial TRFs. PCA represent vectors of variables (locations: *B*-Bhopal, *J*-Jabalpur, *N*-Noida) and factors of TRFs or ribotypes retrieved from the rhizosphere of *Jatropha curcas*. Eigenvalue scaled principal components (PC) 1 represent 46.83% of variation and PC2 represent 31.07% variation. In PCA, *arrows with narrow angles* are strongly correlated; *arrows* that are perpendicular show no correlation and *arrows in opposite* directions indicate negative correlation. More confidence characterizes comparisons between variables with *longer arrows*, as inferences made from variables located near the *center* of the diagram are often imprecise

**Fig. 4:**
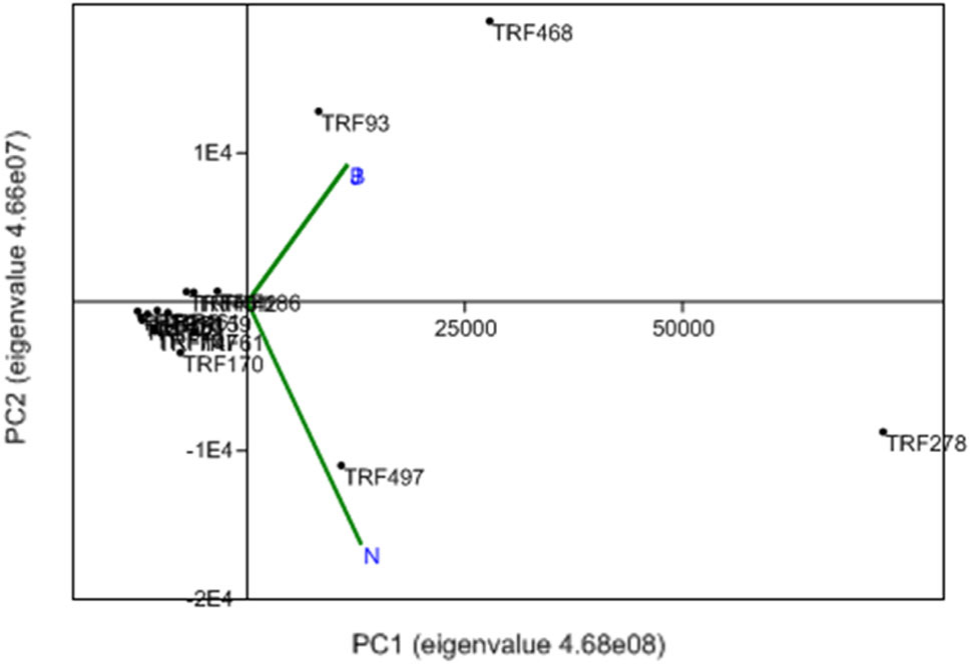
Principal component analysis (PCA) biplot (PC1 and 2) of archaeal TRFs. PCA represent vectors of variables (locations: *B*-Bhopal, *J*-Jabalpur, *N*-Noida) and factors of TRFs or ribotypes retrieved from the rhizosphere of the *Jatropha curcas*. *Eigenvalue* scaled principal components (PC) 1 represent 90.94% of variation and PC2 represent 9.05% variation. In PCA, *arrows with narrow angles* are strongly correlated; *arrows* that are perpendicular show no correlation and *arrows in opposite directions* indicate negative correlation. More confidence characterizes comparisons between variables with *longer arrows*, as inferences made from variables located near the *center* of the diagram are often imprecise

**Fig. 5:**
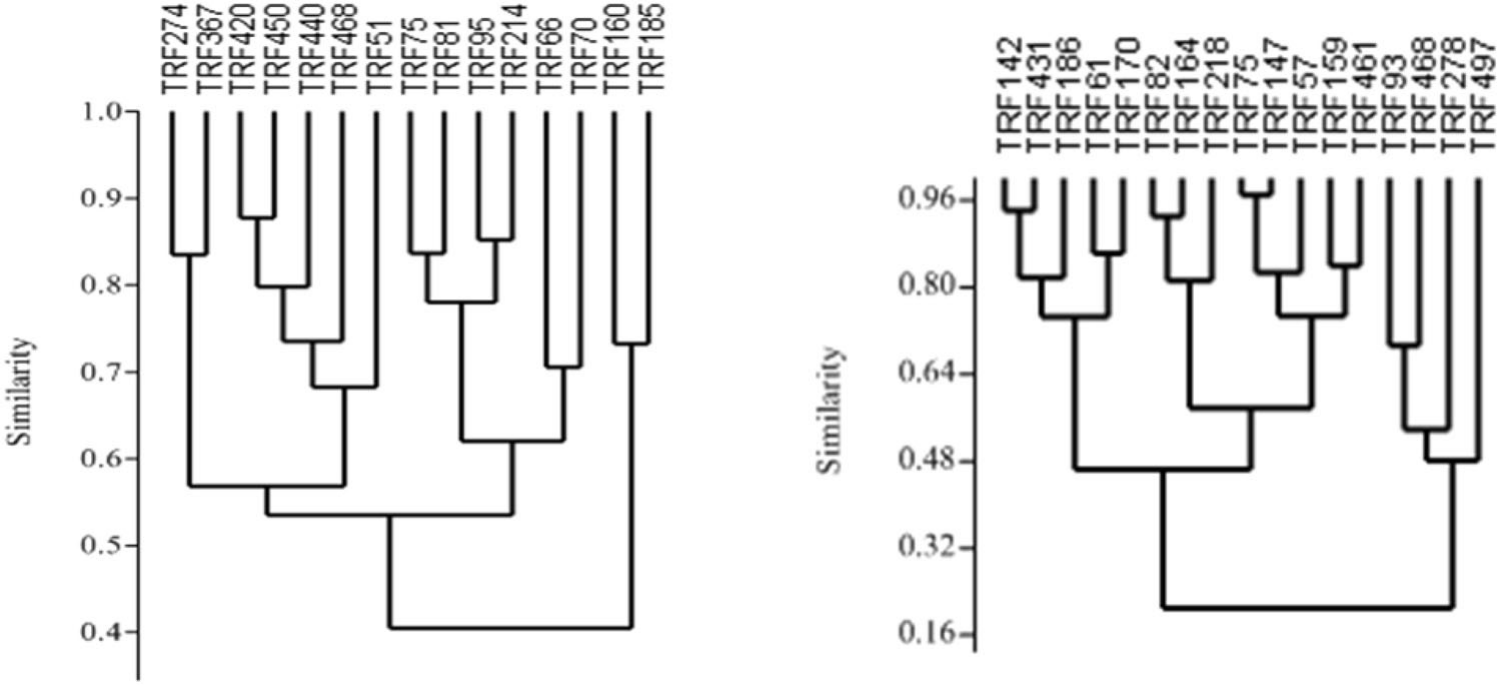
Brays-curtis similarity clustering of bacterial (*left*) and archaeal (*right*) TRFs (ribotypes) retrieved from the rhizosphere of Jatropha curcas. The MDS *plot* gives a 2D representation of relative similarities. TRFs with similar extent of occurrence among the samples are relatively closely spaced

Interactive effect of factors was estimated by the AMMI model (Table 4). Total degree of freedom was 212 for archaea and 152 for bacteria. AMMI model confirmed that the coverage of species by TRFLP was sufficient (sum of squares 1068.60 for bacteria and 959.69 for archaea). Interaction among TRFs and environment (locations) was further assessed by principal component analysis (PCA) with biplot (Fig. 3). PCA of the data matrix resulted in most of the data variance being explained by the first two principal components. For bacteria, PC1 explained 46.83% variation, and 31.07% of variation was rendered by PC2. Bhopal and Noida had similar microbial diversity pattern. TRF51 and TRF420 were dominant in Bhopal and Noida. PCA biplot of archaea indicated that Bhopal and Jabalpur had similar type of community (Fig. 4). TRF93 and TR468 were common in both sites. For archaea, PC1 explained 90.94% of variation and 9.05% of variation by PC2. Bray Curtis similarity grouped TRFs into separate clusters based on the pattern of fluorescence intensity (Fig. 5). Bacterial TRFs formed three clusters. TRF160 and TRF185 formed first cluster. Second cluster was constituted of TRF70, TRF66, TRF214, TRF95, TRF 81, and TRF75. Third cluster was constituted of TRF468, TRF440, TRF450, TRF420, TRF367, and TRF274. Archaea TRFs formed two major clusters. First cluster was constituted of TRF497, TRF278, TRF468, and TRF93 while, all other TRFs constituted second cluster.

**Table 4:** Interaction of factors (TRFs and locations) on microbial diversity

Source	Archaea			Bacteria		
	*df*	SS	MS	*df*	SS	MS
Total	212	3737.90336	17.63162	152	2312.64973	15.21480
TRFs	70	2448.90457	34.98435	50	1341.92820	26.83856
ENVs (location)	2	0.00001	0.00001	2	0.00002	0.00001
T × E	140	1288.99878	9.20713	100	970.72151	9.70722
IPCA 1	71	1068.60875	15.05083	51	959.69960	18.81764
IPCA 2	69	220.39003	3.19406	49	11.02190	0.22494

ANOVA for AMMI model for both archaeal and bacterial TRFs. Model used to determine interactive effect of the factors like TRFs abundance, number and environment

## Discussion

Bacterial species prevalent in rhizosphere of *J. curcas* were mainly belonged to A*ctinobacteria, Verrumicrobia,* and *Firmicutes*. Actinobacteria has been found in rhizosphere of many plants, including cereal crops such as wheat (Zhao et al. 2012; Mingma et al. 2014). *Streptomyces* are major species of *Actinobacteria*, that are commonly found in roots of crops and terrestrial plants (Liu et al. 2015). Actinomycetes has been isolated from the root of the oil-seed plant (Xing et al. 2012). *Nocardioides panzhihuaensis* has been reported from the roots of *J. curcas* (Qin et al. 2012). However, information regarding the prevalence of *Firmicutes* or *Clostridia* in the rhizosphere of *J. curcas* is limited. TRF fragments representing *Verrumicrobia, and Chloroflexi* were retrieved from the rhizosphere of *J. curcas*. In a study, microbial diversity of a native waste-land and planted with *J. curcas* was evaluated. There was a striking increase in the members of Proteobacteria as well as of Bacteroidetes in Jatropha planted soil than the native soil. In contrast, there was a decline in the *Acidobacteria* and *Chloroflexi* community in the fields planted with *J. curcas*.

T-RFLP also indicated the presence of *Bifidobacteria sp, Sphingomonas, Bacillus subtillis*, *Clostridium,* and *Exiguobacterium* in rhizosphere of *J. curcas*. The least abundant species were *Dehalogenomonas, Acidobacteria, Acetobacteria, Verrumicrobia*. Bifidobacteria comes under *Acenitobacter* and exhibits PGPR activities. *Acinetobacter* are plant growth promoters (Huddedar et al. 2002) and they produce phyto hormones (Fiester and Actis 2013), solubilize phosphate (Rokhbakhsh-Zamin et al. 2011), and also produce siderophores (Higgins et al. 2012). *Acinetobacter* suppresses phytopathogenic fungi, such as *Cryphonectria parasitica, Phytophthora capsici,* and *Rhizoctonia solani* (Maindad et al. 2014). TRFs representative of *Sphingomonas* were also found in the rhizospheric samples. These organisms has been found in the rhizosphere of rice (Shahi et al. 2013), apple (Caputo et al. 2015), and ginseng (Singh et al. 2015). *Sphingomonas* promotes plant growth by producing growth hormones, gibberllins and indole acetic acid (Myresiotis et al. 2012). *Bacillus subtilis*, belongs to the phylum *Firmicutes*, has been found in a wide variety of environments and plants. *Bacillus sp* produces phytohormones and exhibits ACC deaminase activity (Bharti et al. 2013). *Exiguobacteria* are gram positive, rod shaped, yellow pigmented bacterium has been found in rhizospheric soil of apple. These bacteria produce siderophore for better nutrient uptake. These strains also produce hydrogen cyanide (HCN) and volatile compounds to protect plants from pathogens like *Rhizoctonia solani, Sclerotium rolfsii, Pythium* and *Fusarium oxysporum* (Selvakumar et al. 2009).

There were 17 archaeal TRFs found in the rhizosphere of *J. curcas* and out of which 11 were affiliated to uncultured archaea. Three TRFs were affiliated to Crenarchaea and other three TRFs were affiliated to Euryarchaea. Archaea has been found in the rhizosphere of different terrestrial plants but mostly were observed in aquatic plants. In temperate grassland pasture, the archaeal communities are mainly *Crenarchaeota* (Nicol et al. 2003). In flooded rice soil ecosystem the ammonia oxidizing archaea are abundant in the rhizosphere than in the bulk soil. The rice cultivation stimulates the abundance of both the nitrifying ammonia oxidizing archaea than ammonia oxidizing bacteria (Chen et al. 2008). A study on the microbial community of three boreal forest tree species (Scots pine, silver birch, and Norway spruce), archael species like methanogenic euryarchaeota belonging to *Methanolobus* sp. and *Methanosaeta* sp. were detected on the roots. In this study the most commonly archaeal 16S rRNA gene sequences were *Crenarchaeota* of 1.1b and 1.1c (Bomberg et al. 2011). Other TRFs represented uncultured archaea like, TRF93 (Crenarchaea), TRF278 (archaea of environmental samples), TRF 468 (Euryarchaea), TRF497 (archaea of environmental samples). Relative abundance of these TRFs followed the trend of TRF 278 > TRF468 > TRF93 > TRF497 (Fig. 2). Crenarchaeota probably were involved in N transformation (nitrification) in rhizosphere of *J. curcas*. However, further research need to be undertaken to verify the role of these archaea on N transformation.

The microbial diversity was evaluated through the Simpson, Shannon and evenness indices (Haegeman et al. 2013). Simpson’s Index (D) measures the probability that two individuals randomly selected from a sample will belong to the same species. Index, 0 represents infinite diversity and 1, no diversity. Bhopal and Noida had high bacterial diversity than Jabalpur. Similar values has been observed in rhizosphere of maize (Pathan et al. 2015). The Shannon index (H) is commonly used to characterize species diversity. It accounts for both abundance and evenness of the species. Similar results have been observed in the Brazilian Atlantic forest soil which is one of the 25 biodiversity hot spots in the world. In these sites the Shannon diversity indices was in the range of 4.12–3.57, ascribed to high microbial diversity (Faoro et al. 2010). Evenness measures relative abundance of the different species. Diversity was high in Bhopal and Noida than Jabalpur. Probably, soil N was related to diversity values. In a study, it was shown that soil with limited inorganic N adversely affected microbial diversity (Thébault et al. 2014). PCA biplot revealed that both Bhopal and Noida possessed similar microbial population. Organic C and total N was high in these soils than Jabalpur. Microbial diversity corresponded with the organic carbon and nitrogen content of soils. Environmental factors that influence microbial community composition and diversity include pH (Eichorst et al. 2007), organic C (Zhou et al. 2002), and available N (Fierer et al. 2003). The expression of microbial activities strongly depends on the organic C and available N content of soil. Soil organic carbon is heterogeneously distributed among different-sized primary particles, forming organo-mineral complexes that respond differently to the plant and environmental factors (Christensen et al. 2001). Microbial diversity of soils varied relatively with the cation exchange capacity (CEC). The capacity of the soil to capture the cations (for example Ca^2+^, Mg^2+^, K^+^, Na^+^, Al^3+^, Fe^2+^, etc.) is called CEC. These cations are held together by the negatively charged clay and organic matter particles in the soil. These cations are easily exchanged with other soil cations and help in microbial metabolism. Probably, CEC favored microbial activity and increased the microbial diversity (Taylor et al. 2002). Clay content of soil reflects organic carbon, CEC and nitrogen (Xue et al. 2013). Therefore, soils with high clay content had high microbial diversity.

Ribotypes like TRF 420 (*Sphingomonas* sp) and TRF51 (*Bifidobacteria*) were dominant in Bhopal and Noida. Whereas, the TRF66 (*Streptococcus*) and TRF70 (*Acinetobacter*) were dominant in Jabalpur. *Actinomycetes* and *Acinetobacter* are prevalent in low nutrient soils. *Acinetobacter* grows well under low nutrient situation and many species form intracellular inclusions of nutrients in the form of polyhydroxy-alkanoates (Abbott et al. 1974). PCA biplot of archaeal TRFs indicated that Bhopal and Jabalpur were closely placed. TRF93 and TRF468 were abundant in Bhopal and Jabalpur samples, while TRF 497, TRF 278 were dominant in the samples collected from Noida. Further studies are essential to explain the linkage between soil properties and archaeal diversity.

Brays curtis algorithm clustered the TRFs into different groups. The clustering depends on fluorescence intensity of the TRFs. If their fluorescence intensity of TRFs varies similarly they are clustered together. For example, TRF 66-TRF 70 and TRF 274-TRF 367 were closely placed. In case of archaea TRF 93, TRF 468, TRF 278, TRF 497 formed one cluster because these TRFs were most dominant. TRF 61, TRF 142, TRF 186, TRF 431 clustered together due to medium range of fluorescence intensity. TRF 57, TRF 82, TRF 164, TRF 218 formed another cluster due to low fluorescence intensity. This clustering strategies has been used to differentiate TRFs of methanotrophs in rice and forest soil (Mohanty et al. 2007). Similar approach has been used to cluster bacterial bph gene in Amazon dark soil to understand microbial diversity (de Lima Brossi et al. 2014). Archaea specific TRF 93, TRF 278, TRF468, TRF 497 were clustered together due to high abundance than other TRFs. Additive main effects and multiplicative interaction model (AMMI) inferred that TRFs were the significant factors than sampling sites. Similar results has been reported in a study determining the microbial diversity in soil during decomposition of Bt rice straw (Lu et al. 2010) and endophytes of potato (Pageni et al. 2013). Here, we show that the structure of bacterial and archaeal communities is not random at the spatial scale because the diversity and composition of bacterial and archaeal community in rhizosphere of *J. curcas* was largely similar. These results suggest that, to some degree, the large scale biogeographical patterns of rhizospheric microorganisms are fundamentally similar for same plant taxa.

To compare bacterial and archaeal diversity and community structure across soils, T-RFLP method was used. This method quantifies sequence variability in the 16S rRNA gene extracted from soil, producing a DNA fingerprint for each microbial community as unique ribotypes (restriction fragments). Although other approaches like sequence analysis of 16S rRNA clone libraries and pyrosequencing provides more detailed phylogenetic information, the T-RFLP method is better suited for analyzing a large number of samples (Burke et al. 2008). It is also commonly used for determining diversity and composition of highly complex microbial communities (Osborne 2014). Major limitation of the T-RFLP method is that it underestimates total bacterial diversity because the method resolves only a limited number of bands per gel (generally <100), and bacterial species can share restriction fragments (Dunbar et al. 2001). However, T-RFLP estimates of microbial community structure have been found to be generally robust and concur with alternative approaches such as 454 pyrosequencing in documenting microbial community differences (Pilloni et al. 2012).

## Conclusion

This study exhibited bacterial and archaeal diversity in rhizosphere of *J. curcas*. Dominant bacterial species were *Actinobacteria, Firmicutes, Acidobacteria, Verrumicrobiaceae,* and *Chlroflexi*. In case of archaea, the dominant species were uncultured *Archaea, Crenarchaea* and *Euarchaea*. Bacterial species detected in the rhizosphere of *J. curcas* were mostly plant growth promoters. Microbial diversity was related to soil C and N content and could be important drivers of the microbial community composition. Although the PGPR activities of the bacterial groups inhabiting the rhizosphere of *J. curcas* are known but literature on archaea is scarce. Further research is warranted to reveal significance of archaea on the growth of *J. curcas*.
